# Atrial GIRK Channels Mediate the Effects of Vagus Nerve Stimulation on Heart Rate Dynamics and Arrhythmogenesis

**DOI:** 10.3389/fphys.2018.00943

**Published:** 2018-07-19

**Authors:** Steven W. Lee, Allison Anderson, Pilar A. Guzman, Atsushi Nakano, Elena G. Tolkacheva, Kevin Wickman

**Affiliations:** ^1^Department of Biomedical Engineering, University of Minnesota, Minneapolis, MN, United States; ^2^Department of Pharmacology, University of Minnesota, Minneapolis, MN, United States; ^3^Department of Integrative Biology and Physiology, University of Minnesota, Minneapolis, MN, United States; ^4^Department of Molecular, Cell, and Developmental Biology, University of California, Los Angeles, Los Angeles, CA, United States

**Keywords:** atrial, vagus nerve stimulation, parasympathetic, arrhythmogenesis, GIRK channel, Kir3

## Abstract

Diminished parasympathetic influence is central to the pathogenesis of cardiovascular diseases, including heart failure and hypertension. Stimulation of the vagus nerve has shown promise in treating cardiovascular disease, prompting renewed interest in understanding the signaling pathway(s) that mediate the vagal influence on cardiac physiology. Here, we evaluated the contribution of G protein-gated inwardly rectifying K^+^ (GIRK/Kir3) channels to the effect of vagus nerve stimulation (VNS) on heart rate (HR), HR variability (HRV), and arrhythmogenesis in anesthetized mice. As parasympathetic fibers innervate both atria and ventricle, and GIRK channels contribute to the cholinergic impact on atrial and ventricular myocytes, we collected *in vivo* electrocardiogram recordings from mice lacking either atrial or ventricular GIRK channels, during VNS. VNS decreased HR and increased HRV in control mice, in a muscarinic receptor-dependent manner. This effect was preserved in mice lacking ventricular GIRK channels, but was nearly completely absent in mice lacking GIRK channels in the atria. In addition, atrial-specific ablation of GIRK channels conferred resistance to arrhythmic episodes induced by VNS. These data indicate that atrial GIRK channels are the primary mediators of the impact of VNS on HR, HRV, and arrhythmogenesis in the anesthetized mouse.

## Introduction

The parasympathetic and sympathetic branches of the autonomic nervous system work antagonistically to maintain cardiovascular homeostasis (Gordan et al., 2015). Autonomic dysregulation, characterized by excessive sympathetic activation and diminished parasympathetic influence, is central to the pathogenesis of cardiovascular diseases including heart failure and hypertension (Bibevski and Dunlap, 2011; Schwartz and De Ferrari, 2011; Mancia and Grassi, 2014). Efferent fibers of the vagus nerve provide the parasympathetic innervation to the heart, and vagus nerve stimulation (VNS) has shown promise in the treatment of cardiovascular diseases, including heart failure and hypertension (Xie et al., 2014; Beaumont et al., 2015; Petkovich et al., 2015; Premchand et al., 2016; Smith et al., 2016). As a result, there is renewed interest in understanding the cell signaling pathways that mediate the impact of VNS on cardiac physiology.

Parasympathetic regulation of heart rate (HR) is mediated via release of acetylcholine (ACh), which activates cardiac muscarinic M_2_ receptors (M_2_R) on sinoatrial and atrioventricular nodal cells, and atrial myocytes (Dhein et al., 2001). M_2_R activation stimulates inhibitory G proteins, which suppress the activity of the HCN (pacemaker or “funny” current, *I*_f_) and L-type Ca^2+^ channels (*I*_Ca,L_) (DiFrancesco et al., 1989; Zaza et al., 1996; DiFrancesco and Borer, 2007; Mangoni and Nargeot, 2008; DiFrancesco, 2010; Kozasa et al., 2018), and activate G protein-gated inwardly rectifying K^+^ (GIRK/Kir3) channels (Logothetis et al., 1987; Wickman et al., 1994; Dhein et al., 2001). The atrial GIRK channel, often referred to as *I*_KACh_, is a heterotetrameric complex composed of homologous subunits – GIRK1/Kir3.1 and GIRK4/Kir3.4 – in 1:1 stoichiometry (Krapivinsky et al., 1995; Corey et al., 1998). Ventricular myocytes also express a GIRK1/GIRK4 channel, albeit at lower levels than in atrial myocytes, and this channel mediates the impact of cholinergic agonists on action potential duration and the excitability of ventricular myocytes (Koumi et al., 1997; Dobrzynski et al., 2001, 2002; Posokhova et al., 2010; Liang et al., 2014; Anderson et al., 2018).

The extent to which the various cardiac conductances (*I*_f_, *I*_Ca,L_, GIRK) contribute to the parasympathetic influence on HR dynamics is unclear. Previous studies investigating the role of GIRK channels have used pharmacological tools to mimic parasympathetic activation in constitutive/global knockout mice lacking either GIRK1 (*Girk1^-/-^*; Bettahi et al., 2002) or GIRK4 (*Girk4^-/-^*; Wickman et al., 1998). Loss of either subunit eliminates the GIRK current in atria and ventricular myocytes (Wickman et al., 1998; Bettahi et al., 2002; Mesirca et al., 2013; Posokhova et al., 2013; Anderson et al., 2018), and yields a comparable attenuation of HR responses to systemic administration of parasympathomimetic agents (Bettahi et al., 2002). For example, experiments involving the administration of the α_1_ adrenergic receptor agonist methoxamine, which evokes a baroreflex-mediated increase in parasympathetic input to the heart, suggested that GIRK channel activation accounts for approximately half of the bradycardic effect of indirect parasympathetic activation in awake mice (Wickman et al., 1998; Bettahi et al., 2002). While direct perfusion of isolated hearts from wild-type and *Girk4^-/-^* mice with relatively high concentrations of ACh (0.3–10 μM) yielded similar results, the bradycardic effect of lower ACh concentrations was comparable in these hearts (Mesirca et al., 2013). Thus, the mediators of parasympathetic influence on HR dynamics may differ depending on the type and intensity of pharmacological stimulation, and the model systems used to evaluate their impact.

While the vagal nerve innervation of atrial and nodal tissue is well-established, vagal innervation of the ventricle has also been reported (Coote, 2013). The relative contribution of atrial and ventricular GIRK channels to the parasympathetic regulation of the heart has not been fully elucidated. Moreover, GIRK1 and GIRK4 are expressed in the hypothalamus, and GIRK4 has been detected specifically in the paraventricular nucleus of the hypothalamus (Karschin et al., 1996; Wickman et al., 2000; Perry et al., 2008; Lujan and Aguado, 2015), a region that regulates cardiac vagal neurons in the brainstem (Pinol et al., 2014; Dyavanapalli et al., 2016). Thus, the impact of *Girk1* or *Girk4* ablation on HR dynamics following pharmacological stimulation of the baroreflex could be due to loss of GIRK channel activity in a central regulator(s) of autonomic function. In this study, we used direct VNS, and new atrial- and ventricle-specific models of GIRK channel ablation, to probe the contribution of cardiac GIRK channels to the parasympathetic regulation of HR, HR variability (HRV), and arrhythmogenesis. Our findings suggest that the impact of direct VNS on cardiac physiology in anesthetized mice is attributable primarily to activation of atrial GIRK channels.

## Materials and Methods

### Animals

All experiments were performed in accordance with the guidelines set forth by the National Institutes of Health Guide for the Care and Use of Laboratory Animals and were approved by the University of Minnesota Institutional Animal Care and Use Committee. C57BL/6J mice and B6.Cg-Gt(ROSA)26Sortm14(CAG-tdTomato)Hze/J (Ai14-tdTomato) reporter mice were purchased from The Jackson Laboratory (Bar Harbor, ME, United States). The generation of constitutive *Girk4^-/-^* mice, as well as mice lacking GIRK1 in ventricular tissue (MLC2VCre(+):*Girk1^fl/fl^*) and their control littermates (MLC2VCre(-):*Girk1^fl/fl^*), was described previously (Wickman et al., 1998; Anderson et al., 2018). To generate mice lacking GIRK1 in atrial tissue, conditional *Girk1* knockout mice (*Girk1^fl/fl^* mice; Marron Fernandez de Velasco et al., 2017) were crossed with an atrial-specific Cre driver line (SLNCre mice; Nakano et al., 2011). SLNCre(+):*Girk1^fl/fl^* mice, and their control littermates (SLNCre(-):*Girk1^fl/fl^*), were used in this study. All mice were housed in a quiet, temperature- and humidity-controlled room with a 12:12 h light-dark cycle. Food and water were available *ad libitum*.

### Microscopy

Mice were injected intraperitoneally (IP) with heparin (250 U) and then anesthetized with ketamine (100 mg/kg) and xylazine (10 mg/kg). Hearts were then excised, fixed, and sectioned as described (Anderson et al., 2018). Once sections were mounted and stained with ProLong Gold Antifade reagent with DAPI (Thermo Fisher Scientific, Waltham, MA, United States), fluorescent images were captured and processed as described (Anderson et al., 2018).

### Cardiomyocyte Culture and Electrophysiology

Adult (8–12 weeks) sinoatrial nodal (SAN) cells and ventricular myocytes were isolated as described (Anderson et al., 2018) and used within 8 h of isolation. Coverslips containing SAN cells or ventricular myocytes were transferred to a perfusion chamber and electrophysiological recordings were conducted as described (Anderson et al., 2018). In brief, whole-cell access was obtained in a bath consisting of (in mM): 130 NaCl, 5.4 KCl, 1 CaCl_2_, 1 MgCl_2_, 5.5 glucose, 5 HEPES/NaOH (pH 7.4). CCh-currents (holding potential of -70 mV) were measured in a high-K^+^ bath solution consisting of (in mM): 120 NaCl, 25 KCl, 1 CaCl_2_, 1 MgCl_2_, 5.5 glucose, 5 HEPES/NaOH (pH 7.4). CCh was applied via ValveLink 8.2 rapid perfusion system (AutoMate Scientific, Berkeley, CA, United States). BaCl_2_ (5 μM) was included in the bath solution for ventricular myocyte recordings to block *I*_K1_, which has been shown to mask GIRK-dependent currents (Beckmann et al., 2008). Studies using these recording conditions, and *Girk1^-/-^* and/or *Girk4^-/-^* mice, have shown definitively that the inward currents evoked by cholinergic agonists are mediated entirely by GIRK channel activation (Bettahi et al., 2002; Posokhova et al., 2013; Anderson et al., 2018). Steady-state CCh-induced currents (pA) were normalized to cell capacitance (pF), and experiments that did not have stable, low access resistances (<20 MΩ) were not included.

### ECG Recordings in SLNCre:*Girk1^fl/fl^* Mice

Male and female SLNCre(-):*Girk1^fl/fl^* and SLNCre(+):*Girk1^fl/fl^* mice (8–12 weeks) were anesthetized with 1.5% isoflurane supplemented with an air mixture of 40% O_2_/60% N_2_ to sustain stable HR. ECG electrodes were placed subcutaneously into the limbs, and ECG data were acquired with an IX-ECG-12 ECG recorder (iWorx Systems, Inc., Dover, NH, United States). Baseline ECG data were recorded for 10 min, at which point the non-selective cholinergic agonist carbachol (CCh) was administered (1.0 mg/kg IP). Baseline (9–10 min) and post-CCh (15–16 min) HR and HRV analysis was performed using Kubios HRV software (Tarvainen et al., 2014). Artifact detection/correction was utilized to detect RR intervals and reduce the impact of ectopic beats and instances of atrioventricular block on HR and HRV analysis.

### Vagus Nerve Bipolar Cuff Electrode Implantation and ECG Recording

Male and female mice (8–12 weeks) were anesthetized with isoflurane (5% for induction and 1.5% for maintenance). After hair removal and skin cleaning, aseptic technique was used to make a ventral midline incision in the neck, and the skin and muscles were retracted. After identifying and isolating the right vagus nerve, a custom helical lead bipolar cuff electrode (Cyberonics, Inc., Houston, TX, United States) was implanted around the nerve (Xie et al., 2014; Lee et al., 2016). The electrode was then connected to the Demipulse Model 103 VNS pulse generator (Cyberonics, Inc.). For VNS experiments, 1 min of baseline ECG was recorded (PRE). Subsequently, VNS (0.25 mA, 10 Hz, and 500 μs) was delivered for 1 min (ON). ECG data were then acquired for 1 min immediately after VNS cessation (POST). Each experiment concluded with PRE, ON, and POST ECG measurements conducted 10 min after administration of atropine (2.0 mg/kg IP).

### Analysis of VNS ECG Recordings

ECG recordings were used to quantify changes in HR and HRV due to VNS throughout the study using Kubios HRV software. All values were averaged over 1 min PRE and ON periods of VNS stimulation. Noisy data segments, premature atrial complexes, and arrhythmic episodes were excluded, and only steady-state data after initial adjustment of the HR to VNS were used for analysis. To account for inter-subject variations in baseline HR, the chronotropic effect of VNS was determined by calculating a relative change in HR (ΔHR_ON_) as follows:

$$\Delta {\text{HR}}_{\text{ON}}=\left(\frac{{\text{HR}}_{\text{ON}}-{\text{HR}}_{\text{PRE}}}{{\text{HR}}_{\text{PRE}}}\right)\times 100\%$$

where HR_ON_ and HR_PRE_ are the mean HR during the VNS ON and PRE periods, respectively. HRV was calculated as the ratio between the standard deviation of RR intervals (SDRR) to mean of RR intervals (mean RR), as described (McIntyre et al., 2014):

$$\text{HRV}=\left(\frac{\text{SDRR}}{\text{Mean RR}}\right)\times 100\%$$

The number of mice that exhibited arrhythmic episodes was also quantified during both PRE and ON periods. An arrhythmic episode was defined as any of the following episodes: (i) skipped beats, (ii) bigeminy, (iii) bradycardia (HR at least 25 bpm less than the mean PRE or ON HR, lasting for more than 2 s), and (iv) tachycardia (HR at least 25 bpm more than the mean PRE or ON HR, lasting for more than 2 s). Premature atrial complexes were not considered arrhythmic episodes as they were observed during VNS regardless of genotype, and frequently occur under healthy conditions (Shindler and Kostis, 2009).

### Statistical Analysis

All data are presented as mean ± SEM. Student’s *t*-test, two-way ANOVA with repeated measures, and Fisher’s exact test were used as appropriate (GraphPad Software, Inc., La Jolla, CA, United States). For studies involving two-way ANOVA analysis, interactions are reported if detected. *Post hoc* analysis (Bonferroni multiple comparison) was used when appropriate. The level of significance was set at *P* < 0.05.

## Results

### Generation and Characterization of an Atrial-Specific *Girk^-/-^* Mouse Line

**FIGURE 1:**
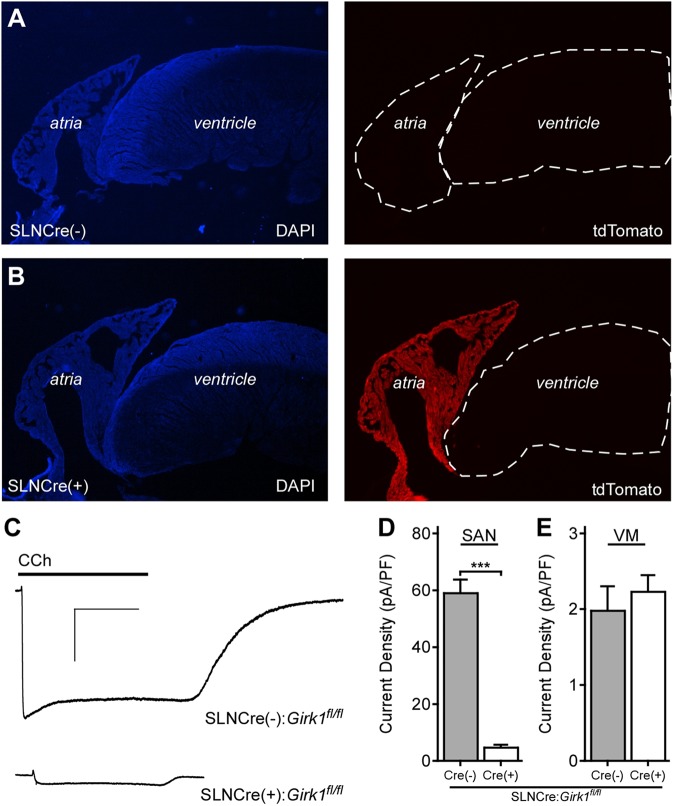
Characterization of an atrial-specific *Girk^-/-^* mouse line. **(A,B)** SLNCre(–) and SLNCre(+) mice were crossed with the Cre-dependent fluorescent reporter strain, Ai14-tdTomato. Representative sections stained with DAPI (Left), and associated tdTomato fluorescence (Right), in hearts from SLNCre(–) (Top) and SLNCre(+) (Bottom) offspring. **(C)** Representative whole-cell currents (*V*_hold_ = –70 mV) evoked by carbachol (CCh, 10 μM) in a high-K^+^ bath solution in adult SAN cells from SLNCre(–):*Girk1^fl/fl^* and SLNCre(+):*Girk1^fl/fl^* mice. Scale bars: 1 nA/10 s. **(D,E)** Summary of CCh-induced currents in adult SAN cells (*t*_47_ = 11.3; ^∗∗∗^*P* < 0.001; *n* = 24–25/genotype) and ventricular myocytes (VM; *t*_21_ = 0.6; *P* = 0.54; *n* = 11–12/genotype) from SLNCre(–):*Girk1^fl/fl^* and SLNCre(+):*Girk1^fl/fl^* mice.

In a recent study, we reported the development and characterization of a ventricle-specific *Girk1^-/-^* mouse (MLC2VCre:*Girk1^fl/fl^*) that lacked ventricular GIRK channel activity (Anderson et al., 2018). To generate an atrial-specific *Girk1^-/-^* line, we crossed conditional *Girk1* knockout mice (*Girk1^fl/fl^*; Marron Fernandez de Velasco et al., 2017) with an atrial-specific Cre driver line (SLNCre; Nakano et al., 2011). The atrial specificity of Cre-dependent recombination in the SLNCre driver line was validated by crossing this line with a Cre-dependent fluorescent reporter mouse (Ai14-tdTomato) (**Figures 1A,B**).

To test whether atrial GIRK channel activity was lost in SLNCre(+):*Girk1^fl/fl^* mice, we measured whole-cell currents evoked by CCh in SAN cells from adult Cre(+) and Cre(-) littermates. CCh-induced current density was significantly smaller in SAN cells from SLNCre(+):*Girk1^fl/fl^* mice when compared to SLNCre(-):*Girk1^fl/fl^* littermates (**Figures 1C,D**). The small residual CCh-induced current seen in SLNCre(+):*Girk1^fl/fl^* SAN cells is likely due to the presence of residual GIRK4 homomeric channels (Corey and Clapham, 1998). Importantly, CCh-induced current densities were comparable in adult ventricular myocytes from SLNCre(+):*Girk1^fl/fl^* and SLNCre(-):*Girk1^fl/fl^* mice (**Figure 1E**).

**FIGURE 2:**
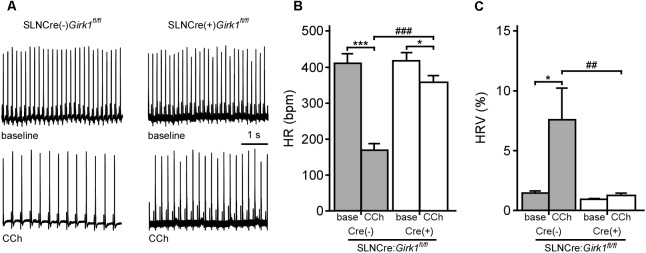
Impact of atrial GIRK channel ablation on HR and HRV responses to carbachol. **(A)** Representative segments of ECG recordings from anesthetized SLNCre(–):*Girk1^fl/fl^* and SLNCre(+):*Girk1^fl/fl^* mice, at baseline (Top) and following administration of CCh (1.0 mg/kg IP; Bottom). **(B)** Summary of HR data at baseline (base) and after CCh injection for SLNCre(–):*Girk1^fl/fl^* (*n* = 7) and SLNCre(+):*Girk1^fl/fl^* (*n* = 8) mice. Two-way ANOVA analysis revealed an interaction between genotype and treatment (*F*_1,13_ = 38.6; *P* < 0.001). Symbols: ^∗^*P* < 0.05 and ^∗∗∗^*P* < 0.001, vs. baseline (within genotype); ^###^*P* < 0.001 vs. SLNCre(–):*Girk1^fl/fl^* (within treatment). **(C)** Summary of HRV data at baseline (base) and after CCh injection for SLNCre(–):*Girk1^fl/fl^* (*n* = 7) and SLNCre(+):*Girk1^fl/fl^* (*n* = 8) mice. HRV was calculated as the ratio of standard deviation of RR to mean RR (see section “Materials and Methods”). Two-way ANOVA analysis revealed an interaction between genotype and treatment (*F*_1,13_ = 5.4; *P* < 0.05). Symbols: ^∗^*P* < 0.05 vs. baseline (within genotype); ^##^*P* < 0.01 vs. SLNCre(–):*Girk1^fl/fl^* mice (within treatment).

Constitutive *Girk1^-/-^* and *Girk4^-/-^* mice exhibit blunted HR and HRV responses to cholinergic agonists (Wickman et al., 1998; Kovoor et al., 2001; Bettahi et al., 2002; Anderson et al., 2018). To discern whether these effects are mediated by activation of atrial GIRK channels, we recorded ECGs in anesthetized SLNCre(-):*Girk1^fl/fl^* and SLNCre(+):*Girk1^fl/fl^* mice, before and after administration of CCh (1.0 mg/kg IP) (**Figure 2A**). We observed no differences in baseline HR or HRV between SLNCre(-):*Girk1^fl/fl^* and SLNCre(+):*Girk1^fl/fl^* mice (**Figures 2B,C**). CCh-induced bradycardia, while present, was smaller in SLNCre(+):*Girk1^fl/fl^* mice than in SLNCre(-):*Girk1^fl/fl^* littermates (**Figure 2B**). In addition, the CCh-induced increase in HRV in control SLNCre(-):*Girk1^fl/fl^* mice was absent in SLNCre(+):*Girk1^fl/fl^* mice (**Figure 2C**). Thus, mice lacking atrial GIRK channels exhibit diminished HR and absent HRV responses to CCh challenge, reminiscent of phenotypes reported in *Girk1^-/-^* and *Girk4^-/-^* mice.

### Impact of Whole-Heart and Tissue-Specific GIRK Channel Ablation on Baseline HR and HRV

**FIGURE 3:**
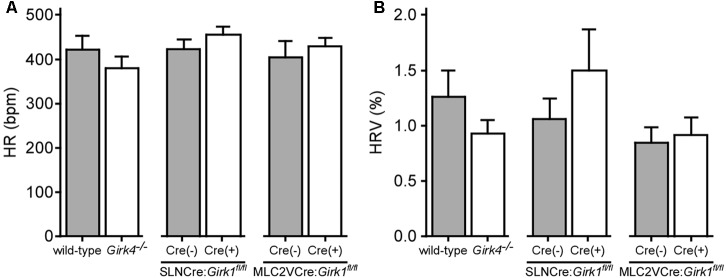
Impact of whole-heart and tissue-specific GIRK channel ablation on baseline HR and HRV. **(A)** Summary of baseline HR data. There were no significant differences in baseline HR between wild-type (*n* = 10) and *Girk4^-/-^* (*n* = 10) mice (*t*_18_ = 1.0; *P* = 0.32), SLNCre(–):*Girk1^fl/fl^* (*n* = 6) and SLNCre(+):*Girk1^fl/fl^* (*n* = 5) mice (*t*_9_ = 1.1; *P* = 0.29), or MLC2VCre(–):*Girk1^fl/fl^* (*n* = 6) and MLC2VCre(+):*Girk1^fl/fl^* (*n* = 8) mice (*t*_12_ = 0.7; *P* = 0.51). **(B)** Summary of baseline HRV data. There were no significant differences in HRV between wild-type (*n* = 10) and *Girk4^-/-^* (*n* = 10) mice (*t*_18_ = 1.2; *P* = 0.23), SLNCre(–):*Girk1^fl/fl^* (*n* = 5) and SLNCre(+):*Girk1^fl/fl^* (*n* = 5) mice (*t*_8_ = 1.1, *P* = 0.32), or MLC2VCre(–):*Girk1^fl/fl^* (*n* = 6) and MLC2VCre(+):*Girk1^fl/fl^* (*n* = 8) mice (*t*_12_ = 0.32; *P* = 0.76).

We next evaluated HR and HRV prior to and during acute VNS in anesthetized SLNCre(+):*Girk1^fl/fl^* and SLNCre(-):*Girk1^fl/fl^* mice, and MLC2VCre(+):*Girk1^fl/fl^* and MLC2VCre(-):*Girk1^fl/fl^* mice. These studies also involved wild-type and constitutive *Girk4^-/-^* mice, which lack cardiac GIRK channels (whole-heart ablation). We did not observe a difference in baseline HR or HRV between wild-type and *Girk4^-/-^* mice (**Figures 3A,B**). Similarly, there were no significant differences in baseline HR and HRV between SLNCre(-):*Girk1^fl/fl^* and SLNCre(+):*Girk1^fl/fl^* mice, or MLC2VCre(-):*Girk1^fl/fl^* and MLC2VCre(+):*Girk1^fl/fl^* mice (**Figures 3A,B**).

### Whole-Heart and Tissue-Specific GIRK Channel Ablation and VNS-Induced Bradycardia

**FIGURE 4:**
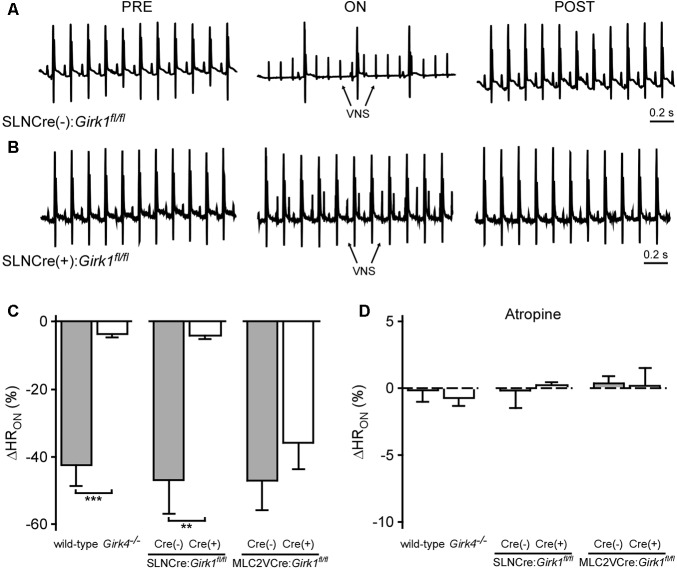
Impact of whole-heart and tissue-specific GIRK channel ablation on VNS-induced decrease in HR. **(A,B)** Representative ECG segments from anesthetized SLNCre(–):*Girk1^fl/fl^* (Top) and SLNCre(+):*Girk1^fl/fl^* (Bottom) mice prior to (PRE, Left), during (ON, Middle), and after (POST, Right) VNS. The small spikes observed during VNS (ON) correspond to electrical stimulation artifacts. **(C)** Relative change in HR (ΔHR_ON_) during VNS. A significant difference in ΔHR_ON_ was observed between wild-type (*n* = 9) and *Girk4^-/-^* (*n* = 10) mice (*t*_17_ = 6.5; ^∗∗∗^*P* < 0.001), as well as SLNCre(–):*Girk1^fl/fl^* (*n* = 6) and SLNCre(+):*Girk1^fl/fl^* (*n* = 5) mice (*t*_9_ = 3.9; ^∗∗^*P* < 0.01). No difference was seen between MLC2VCre(–):*Girk1^fl/fl^* (*n* = 6) and MLC2VCre(+):*Girk1^fl/fl^* (*n* = 8) mice (*t*_12_ = 0.95; *P* = 0.36). **(D)** Relative change in HR (ΔHR_ON_) during VNS, in the presence of atropine (2.0 mg/kg IP). There were no significant differences between wild-type (*n* = 4) and *Girk4^-/-^* (*n* = 6) mice (*t*_8_ = 0.6; *P* = 0.59), SLNCre(–):*Girk1^fl/fl^* (*n* = 6) and SLNCre(+):*Girk1^fl/fl^* (*n* = 4) mice (*t*_8_ = 0.2; *P* = 0.82), and MLC2VCre(–):*Girk1^fl/fl^* (*n* = 6) and MLC2VCre(+):*Girk1^fl/fl^* (*n* = 4) mice (*t*_8_ = 0.2, *P* = 0.88).

We next assessed the impact of whole-heart or tissue-specific GIRK channel ablation on the bradycardic effects of acute VNS, by comparing the relative change in HR evoked by VNS (ΔHR_ON_). Acute VNS suppressed HR by more than 40% in anesthetized wild-type mice, but had little impact on HR in *Girk4^-/-^* mice (**Figure 4C**). SLNCre(+):*Girk1^fl/fl^* mice also displayed minimal VNS-induced bradycardia relative to SLNCre(-)*:Girk1^fl/fl^* controls (**Figures 4A–C**). In contrast, the VNS-induced decrease in HR was comparable between MLC2VCre(+):*Girk1^fl/fl^* and MLC2VCre(-):*Girk1^fl/fl^* mice (**Figure 4C**). After VNS was terminated, HR values returned to baseline for all genotypes (data not shown). To determine if the bradycardic effect of VNS was dependent on muscarinic receptor activation, we also evaluated the impact of VNS on HR following administration of atropine (2.0 mg/kg IP). In the presence of atropine, VNS had no effect on HR in any of the genotypes evaluated (**Figure 4D**). Collectively, these findings suggest that VNS-induced bradycardia in anesthetized mice is mediated predominantly by the muscarinic receptor-dependent activation of atrial GIRK channels.

### Whole-Heart and Tissue-Specific GIRK Channel Ablation and VNS-Induced Increase in HRV

**FIGURE 5:**
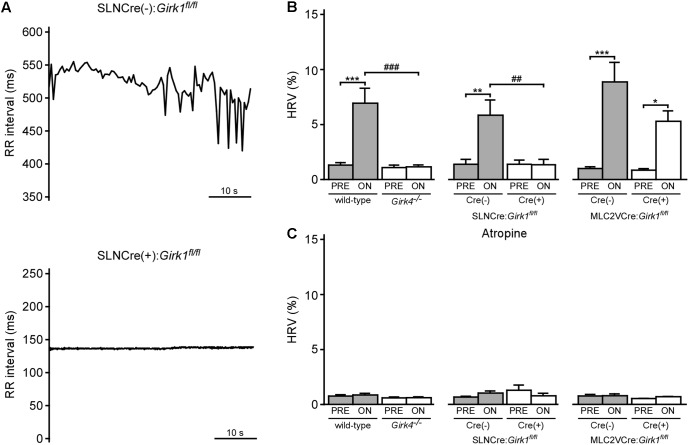
Impact of whole-heart and tissue-specific GIRK channel ablation on VNS-induced increase in HRV. **(A)** Typical RR tachograms extracted from ECG recordings while VNS was on, comparing beat-to-beat changes in RR intervals between SLNCre(–):*Girk1^fl/fl^* (Top) and SLNCre(+):*Girk1^fl/fl^* (Bottom) mice. **(B)** Summary of mean HRV data at baseline and during VNS. Two-way ANOVA analysis revealed an interaction between genotype and treatment between wild-type (*n* = 8) and *Girk4^-/-^* (*n* = 10) mice (*F*_1,16_ = 20.3; *P* < 0.001), and between SLNCre(–):*Girk1^fl/fl^* (*n* = 6) and SLNCre(+):*Girk1^fl/fl^* (*n* = 5) mice (*F*_1,9_ = 12.8; *P* < 0.01). There was a significant main effect of treatment (*F*_1,12_ = 44.0; *P* < 0.001) between MLC2VCre(–):*Girk1^fl/fl^* (*n* = 6) and MLC2VCre(+):*Girk1^fl/fl^* (*n* = 8) littermates, but no main effect of genotype (*F*_1,12_ = 4.2; *P* = 0.06) or interaction between genotype and treatment (*F*_1,12_ = 3.2; *P* = 0.10). Symbols: ^∗^*P* < 0.05, ^∗∗^*P* < 0.01, and ^∗∗∗^*P* < 0.001, vs. PRE (within genotype); ^###^*P* < 0.001, wild-type vs. *Girk4^-/-^* (within treatment); ^##^*P* < 0.01, SLNCre(–):*Girk1^fl/fl^* vs. SLNCre(+):*Girk1^fl/fl^* (within treatment). **(C)** Summary of mean HRV data at baseline and during VNS, following atropine administration. In wild-type (*n* = 4) and *Girk4^-/-^* (*n* = 6) mice, there was a main effect of treatment (*F*_1,8_ = 5.7, *P* < 0.05), however, *post hoc* analysis did not reveal any significance within either genotype. There was no main effect of genotype (*F*_1,8_ = 2.0, *P* = 0.19), or interaction between genotype and treatment (*F*_1,8_ = 3.2; *P* = 0.11), in wild-type and *Girk4^-/-^* mice as well. There was no main effect of treatment (*F*_1,8_ = 0.1, *P* = 0.76) or genotype (*F*_1,8_ = 0.53, *P* = 0.49), and no interaction between treatment and genotype (*F*_1,8_ = 3.5, *P* = 0.1), in SLNCre(–):*Girk1^fl/fl^* (*n* = 6) and SLNCre(+):*Girk1^fl/fl^* (*n* = 4) mice. There was no main effect of treatment (*F*_1,7_ = 0.45, *P* = 0.52) or genotype (*F*_1,7_ = 0.79, *P* = 0.40), and no interaction between treatment and genotype (*F*_1,7_ = 0.3; *P* = 0.62), in MLC2VCre(–):*Girk1^fl/fl^* (*n* = 6) and MLC2VCre(+):*Girk1^fl/fl^* (*n* = 4) mice.

We next examined the impact of VNS on HRV in whole-heart and tissue-specific *Girk^-/-^* mice. VNS evoked a significant increase in HRV in wild-type and SLNCre(-):*Girk1^fl/fl^* mice, and this effect was absent in both constitutive *Girk4^-/-^* and SLNCre(+):*Girk1^fl/fl^* mice (**Figures 5A,B**). In contrast, the increase in HRV induced by VNS was similar in both MLC2VCre(-):*Girk1^fl/fl^* and MLC2VCre(+):*Girk1^fl/fl^* mice (**Figure 5B**). Atropine blocked the VNS-induced increase in HRV in wild-type, SLNCre(-):*Girk1^fl/fl^*, MLC2VCre(-):*Girk1^fl/fl^*, and MLC2VCre(+):*Girk1^fl/fl^* mice (**Figure 5C**). Thus, the VNS-induced increase in HRV in mice appears to be mediated predominantly through the muscarinic receptor-dependent activation of atrial GIRK channels.

### Whole-Heart and Tissue-Specific GIRK Channel Ablation and VNS-Induced Arrhythmias

**FIGURE 6:**
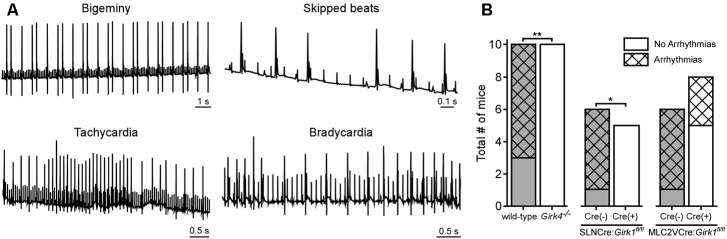
Impact of whole-heart and tissue-specific GIRK channel ablation on VNS-induced arrhythmogenesis. **(A)** Representative segments of ECG recordings of arrhythmias (bigeminy, skipped beats, bradycardia, and tachycardia) observed during VNS. **(B)** Total number of mice exhibiting arrhythmic episodes during VNS. Fisher’s exact test revealed a significant difference in arrhythmia incidence between wild-type (*n* = 10) and *Girk4^-/-^* (*n* = 10) mice (^∗∗^*P* < 0.01), and SLNCre(–):*Girk1^fl/fl^* (*n* = 6) and SLNCre(+):*Girk1^fl/fl^* (*n* = 5) mice (^∗^*P* < 0.05), but no difference between MLC2VCre(–):*Girk1^fl/fl^* (*n* = 6) and MLC2VCre(+):*Girk1^fl/fl^* (*n* = 8) mice (*P* = 0.14).

Direct VNS has been utilized extensively in previous studies to induce and maintain arrhythmias in various large mammalian species (Zhang and Mazgalev, 2011). To probe the involvement of GIRK channel activation on VNS-induced arrhythmogenesis, we quantified arrhythmic episodes prior to and after VNS in whole-heart and tissue-specific *Girk^-/-^* mice, and their respective controls (**Figure 6**). No mice displayed arrhythmic episodes prior to VNS, nor were arrhythmic episodes seen in mice given atropine (data not shown). Arrhythmic episodes were frequently observed, however, during VNS in wild-type and SLNCre(-):*Girk1^fl/fl^* mice (**Figure 6B**). In contrast, *Girk4^-/-^* and SLNCre(+):*Girk1^fl/fl^* mice did not exhibit VNS-induced arrhythmias (**Figure 6B**). VNS-induced arrhythmogenesis was comparable in MLC2VCre(+):*Girk1^fl/fl^* and MLC2VCre(-):*Girk1^fl/fl^* mice. Overall, these findings suggest that atrial GIRK channels play a critical role in the induction of arrhythmias during VNS.

## Discussion

Parasympathetic regulation of cardiac physiology is thought to involve muscarinic-dependent and independent actions on atrial and ventricular tissue, and multiple enzymes and ion channels (Coote, 2013; Gordan et al., 2015). While previous studies have suggested that GIRK channels, *I*_Ca,L_, and *I*_f_ mediate the muscarinic receptor-dependent effects of parasympathetic activation on the heart, their relative contributions remain unclear. Here, we demonstrate that the impact of direct VNS on HR, HRV, and arrhythmogenesis in anesthetized mice is mediated primarily by the muscarinic receptor-dependent activation of atrial GIRK channels. Notably, this study is the first to evaluate the impact of GIRK channel ablation, either in whole-heart or in tissue-specific fashion, on the effects of direct VNS in mice.

Constitutive *Girk1^-/-^* and *Girk4^-/-^* mice display blunted HR and HRV responses to pharmacologic treatments that stimulate or mimic parasympathetic activity (Wickman et al., 1998; Kovoor et al., 2001; Bettahi et al., 2002; Mesirca et al., 2013). However, the degree of bradycardia conferred by GIRK channel activation that has been reported in these studies has varied. For example, the bradycardic effect evoked by methoxamine activation of the baroreflex was ∼50% smaller in *Girk4^-/-^* mice (Wickman et al., 1998; Bettahi et al., 2002), whereas experiments involving direct perfusion of ACh onto SA nodal tissue, or of isolated mouse hearts, have suggested that the GIRK channel contribution to the bradycardic effect of cholinergic agonists is concentration-dependent (Mesirca et al., 2013). Consistent with this premise, DiFrancesco et al. (1989) showed a concentration-dependent effect of ACh in modulation of *I*_f_ and *I*_KACh_ in SAN cells: whereas nanomolar concentrations of ACh inhibited *I*_f_, 20-fold higher concentrations were required to activate *I*_KACh_.

In this study, we found that nearly all of the HR and HRV response to electrical stimulation of the vagus nerve was absent in constitutive *Girk4^-/-^* mice, and in mice lacking only atrial GIRK channels. Atropine blocked VNS-induced HR and HRV responses, indicating that these effects are dependent on muscarinic receptor activation. While the prominent contribution of GIRK channels revealed in this study could reflect our utilization of a relatively strong VNS protocol, or the fact that all recordings were made in anesthetized mice, it was nevertheless surprising that minimal residual impact of VNS stimulation on HR or HRV was observed in *Girk4^-/-^* mice and SLNCre(+):*Girk1^fl/fl^* mice. Given that *I*_Ca,L_ and *I*_f_ conductances have been recorded in SAN cells from *Girk4^-/-^* mice (Mesirca et al., 2013), and that residual cholinergic-induced bradycardia was seen in *Girk4^-/-^* mice (Wickman et al., 1998; Bettahi et al., 2002; Mesirca et al., 2013; Anderson et al., 2018), we do not believe that the lack of VNS-induced effects in *Girk4^-/-^* or SLNCre(+):*Girk1^fl/fl^* mice reflects a loss of muscarinic receptor expression and/or function. Notably, our findings are consistent with those of a recent study showing that while knockdown of HCN4 exaggerated the bradycardic response to VNS in mice, over-expression of HCN4 only blunted VNS-induced bradycardia in the presence of isoproterenol (Kozasa et al., 2018). Thus, muscarinic-dependent activation of atrial GIRK channels appears to play the primary role in the effect of VNS on HR dynamics, at least in the anesthetized mouse.

The VNS-induced reduction of atrial effective refractory period and increase in heterogeneity throughout the atria has been linked to atrial arrhythmogenesis (Prystowsky et al., 1983; Zhang and Mazgalev, 2011; Shen and Zipes, 2014). Indeed, VNS is frequently utilized for the induction and maintenance of atrial fibrillation in large mammalian species (Katsouras et al., 2009; Zhang and Mazgalev, 2011). Additionally, high vagal tone is associated with various bradyarrhythmias, including atrioventricular block and sinus node dysfunction, in human patients (Aksu et al., 2018). Here, we demonstrated that VNS is also pro-arrhythmic in the mouse, as arrhythmic episodes were only observed during VNS. Moreover, the arrhythmogenic effect of VNS in the anesthetized mouse is primarily attributable to the muscarinic receptor-dependent activation of atrial GIRK channels.

VNS has been shown to exhibit anti-inflammatory effects by decreasing pro-inflammatory and/or increasing anti-inflammatory cytokine levels in various animal models (Zhang et al., 2009; Calvillo et al., 2011; Mihaylova et al., 2012). Thus, it will be interesting to test whether VNS confers a therapeutic benefit in heart diseases characterized by pro-inflammatory cytokines (Hedayat et al., 2010). In addition, although GIRK channel activation appears to mediate most of the VNS-induced influence on HR dynamics, further studies are needed to discern whether VNS-induced activation of atrial GIRK-channels underlies its cardioprotective benefit in diseases such as heart failure. As part of this effort, experiments should be conducted to determine whether it is possible to identify VNS parameters that deliver therapeutic benefits without promoting arrhythmogenesis.

Notably, neither *Girk4^-/-^* nor SLNCre(+):*Girk1^fl/fl^* mice exhibited arrhythmic episodes during VNS. These findings are consistent with a previous report showing that *Girk4* ablation confers resistance to pacing-induced atrial fibrillation in mice (Kovoor et al., 2001), and can restore normal cardiac rhythm in mouse models of sick sinus syndrome and AV block (Mesirca et al., 2014, 2016). Interestingly, a recent study reported that increased GIRK channel activity secondary to a mutation in the gene encoding the G protein β2 subunit (Gβ2; *GNB2*) is associated with AV nodal dysfunction in humans (Stallmeyer et al., 2017). Collectively, these insights highlight the clinical potential of pharmacologic or genetic suppression of atrial GIRK channels for the treatment of supraventricular arrhythmias.

In summary, we conclude that the muscarinic receptor-dependent activation of atrial GIRK channels is the dominant mediator of VNS-induced effects on HR, HRV, and arrhythmogenesis in mice. Our findings highlight the potential therapeutic benefit of selectively targeting this branch of the parasympathetic signaling for the management of atrial arrhythmias.

## Data Availability

Datasets are available on request.

## Author Contributions

SWL, AA, ET, and KW were responsible for the conception of this study, wrote and prepared the manuscript. All authors contributed to experimental design, execution of experiments, and data analysis. All authors reviewed the manuscript.

## Conflict of Interest Statement

The authors declare that the research was conducted in the absence of any commercial or financial relationships that could be construed as a potential conflict of interest.
